# Modulating charge-dependent and folding-mediated antimicrobial interactions at peptide–lipid interfaces

**DOI:** 10.1007/s00249-016-1180-8

**Published:** 2016-11-10

**Authors:** Patrizia Iavicoli, François Rossi, Baptiste Lamarre, Angelo Bella, Maxim G. Ryadnov, Luigi Calzolai

**Affiliations:** 1 European Commission, DG Joint Research Centre, Via Enrico Fermi, 2749, Ispra, VA 21027 Italy; 2 National Physical Laboratory, Teddington, TW11 0LW UK

**Keywords:** Antimicrobial peptides, Synthetic membranes, Dynamic light scattering, Circular dichroism, Fluorescence, Nuclear magnetic resonance

## Abstract

Peptide–lipid interactions support a variety of biological functions. Of particular interest are those that underpin fundamental mechanisms of innate immunity that are programmed in host defense or antimicrobial peptide sequences found virtually in all multicellular organisms. Here we synthetically modulate antimicrobial peptide–lipid interactions using an archetypal helical antimicrobial peptide and synthetic membranes mimicking bacterial and mammalian membranes in solution. We probe these interactions as a function of membrane-induced folding, membrane stability and peptide–lipid ratios using a correlative approach encompassing light scattering and spectroscopy measurements such as circular dichroism spectroscopy, fluorescence and nuclear magnetic resonance spectroscopy. The peptide behavior is assessed against that of its anionic counterpart having similar propensities for α-helical folding. The results indicate strong correlations between peptide folding and membrane type, supporting folding-responsive binding of antimicrobial peptides to bacterial membranes. The study provides a straightforward approach for modulating structure–activity relationships in the context of membrane-induced antimicrobial action, thus holding promise for the rational design of potent antimicrobial agents.

## Introduction

Antimicrobial peptides (AMPs) are universal effector molecules of innate immunity found in all multicellular organisms (Brogden 2005). Although their mode of action is not entirely understood, it is believed to be fundamentally the same for more than 1000 AMPs reported to date (Fjell et al. 2007; Sato and Feix 2006; Sang and Blecha 2008). AMPs, most of which are cationic, recognize and bind to anionic microbial membranes, whereupon they fold as amphipathic structures whose hydrophobic faces incorporate into phospholipid bilayer interfaces causing membrane disruption and collapse. Acquiring resistance against such formidable membrane-active agents is an extremely high price for microorganisms to pay. Therefore, it is not surprising that the peptides are increasingly considered as next-generation antibiotics (Fjell et al. 2012; Giuliani and Rinaldi 2011). As fundamental as their mode of action can be, AMPs largely rely on peptide–lipid interactions. The nature and extent of such interactions define specific mechanisms of membrane disintegration including carpet-like and poration (barrel-stave, toroidal) scenarios. The preference of one mechanism over another appears to depend on several factors, mainly structural features programmed in peptide sequences and how these features respond to lipid binding. In this regard, it is important to develop a thorough understanding of the structure–activity relationships of membrane-associated AMPs, which may also underpin the development of more powerful and efficient alternatives to conventional antibiotics. In this study, we applied a correlative measurements approach to probe antimicrobial peptide–lipid interactions using synthetic mimetics of bacterial and mammalian membranes and an archetypal helical AMP. The activity of the AMP was assessed against that of its anionic counterpart, which has a similar propensity for helical folding, but which lacks the ability to fold in the presence of phospholipid membranes and is therefore biologically inactive. We probe the interactions of both peptides with lipid membranes by low- and high-resolution methods—circular dichroism (CD) spectroscopy, fluorescence and ^1^H-NMR spectroscopy, respectively.

## Materials and methods

### Materials

Dilauroyl phosphatidylcholine (DLPC), dilauroyl phosphatidylglycerol (DLPG), palmitoyl oleoyl phosphatidylcholine (POPC), and palmitoyl oleoyl phosphatidylglycerol (POPG) were purchased from Avanti^®^ Polar Lipids, Inc. Monosodium phosphate and disodium phosphate were purchased from Sigma-Aldrich.

### Liposome preparation

Unilamellar vesicles assembled from POPC, POPC/POPG (3:1 mol ratio), DLPC or DLPC/DPLG (3:1 mol ratio) were prepared by dissolving the lipids in a chloroform:methanol (2:1, v:v) mixture at 10 mg/ml. The solution was then evaporated under nitrogen flow overnight, and the obtained dried film was re-suspended in 10 mM phosphate buffer, pH 7.4. The preparation was vortexed for 2 min and sonicated at room temperature for 2 min. The cycle was repeated three times. The solution was extruded 21 times through a 100-nm polycarbonate membrane before each use.

### Peptide synthesis

The peptides were assembled on a Liberty microwave peptide synthesizer (CEM Corp.) as peptide amides using Rink Amide MBHA resin, standard solid-phase Fmoc/tBu protocols and HBTU/DIPEA as coupling reagents. Following cleavage and deprotection (95% TFA, 2.5% TIS, 2.5% water), peptides were purified using RP-HPLC and their purities were confirmed by analytical RP-HPLC and MALDI-ToF mass spectrometry (Bruker Daltonics Ltd, UK) with α-cyano-4-hydroxycinnamic acid as the matrix.

MS [M + H]^+^: (+)-helix, *m*/*z* 2537.2 (calc), 2537.4 (found); (−)-helix, *m*/*z* 2371.2 (calc), 2370.9 (found); BMAP-27-*m*/*z* 3282.2 (calc), 3283.2 (found); cecropin B-*m*/*z* 3834.5 (calc.), 3836.0 (found).

### Preparation of lipid to peptide (L/P) molar ratios

(+)-helix and (−)-helix peptide solutions at 0.5 mM were prepared fresh before each experiment in 10 mM phosphate buffer, pH 7.4. The concentration was calculated by reading UV–Vis absorbance at 280 nm. For all experiments, the lipid concentration of liposomes was kept constant at 1.2 mM for all liposome–peptide complexes studied. For a desired L/P ratio (mole), a 250-µl peptide aliquot at a defined concentration (from 120 to 16 µM) was added immediately before the measurements to 750 µl of a 1.6-mM liposome solution. The final volume of each sample solution was 1 ml.

### Dynamic light scattering

The hydrodynamic diameter and the Z-potential were measured using a Malvern Zetasizer Nano-ZS instrument. Each sample has a volume of 500 µl in a PMMA cuvette and each measurement was done at 25 °C in triplicate. The refractive index of liposomes used was 1.47, the absorption coefficient was 0.001 (Malvern database). The number of data sets of each measurement was automatically optimized by the instrument according to the quality of the sample and the intensity of the scattered light (ideal correlogram amplitude of 0.8). The data were analyzed by single exponential cumulant analysis using the manufacturer’s Dispersion Technology Software (DTS version 7.01) that provides the Z average (i.e., the intensity-weighted mean hydrodynamic size of the ensemble of particles) and the polydispersity index, PdI, which accounts for peak broadening effects. In the case of a Gaussian distribution, PdI = (width of distribution/mean size)^2^. Thus, a perfectly monodispersed sample would have PdI equal to zero. The instrument was calibrated using varied sizes of polystyrene latex beads standards.

### Circular dichroism spectroscopy

CD measurements were performed using a Jasco J-815 spectropolarimeter. The instrument was purged with oxygen-free nitrogen for at least 30 min prior to spectra acquisition. The temperature of the cell compartment was equilibrated to 25 °C using a temperature control unit. Spectra in the 185–260-nm wavelength range were collected by acquiring and averaging a minimum of four scans. Data points for CD spectra were recorded at a 1-nm interval using a 1-nm bandwidth, with a 1-s response time and 50-nm/min scanning speed.

Spectra of bare liposomes at different concentrations were run as blanks to be subtracted from peptide to liposome spectra. All measurements were taken in ellipticities in mdeg and after baseline correction were converted to mean residue ellipticities (MRE: deg cm^2^ dmol^−1^) by normalizing for the concentration of peptide bonds and cuvette pathlength.

### Fluorescence spectroscopy

The spectra were measured using a Agilent Cary Eclipse Fluorescence Spectrophotometer (Agilent Technologies). The fluorescence was excited at 274 nm and emission was scanned from 280 to 400 nm. The excitation and emission slits were 5 nm.

### Nuclear magnetic resonance spectroscopy

Samples for NMR experiments were prepared by transferring 450 µl of sample into NMR tubes and adding 20 µl D_2_O. For the measurements of the peptides in TFE, 50% deuterated TFE was used. ^1^H NMR experiments were collected with a 500-MHz Bruker instrument using an excitation sculpting pulse sequence for water suppression (acquisition time of 1 s and relaxation delay of 2 s) and 256 or 2024 scans depending on the concentration of the sample. Data were processed with the same processing parameters for all samples using the TopSpin NMR software. An exponential function with a line broadening of 1 Hz was applied to each data set, before Fourier transformation.

Minimum inhibitory concentrations (MICs) were determined by broth microdilution according to the protocols by Clinical and Laboratory Standards Institute; 100 μl of 0.5–1 × 106 CFU per ml of each bacterium (Table 1) in Mueller–Hinton media broth (Oxoid) were incubated with 100 μl of serial twofold peptide dilutions in 96-well microtiter plates on a 3D orbital shaker. The absorbance was measured after peptide addition at 600 nm using a Victor 2 plate reader (Perkin-Elmer). MICs were defined as the lowest peptide concentration after 24 h at 37 °C. All tests were done in triplicate.

**Table 1:** Biological activities of the peptides used in the study

Cell	Minimum inhibitory concentrations, µM			
	(+)-helix	(−)-helix	Cecropin B	Cathelicidin^a^
*P. aeruginosa* (ATCC27853)	4	≫250	1.5	4.5
*S. aureus* (ATCC6538)	9	≫250	>100	>100
*E. coli* (K12)	10	≫250	<1	3
	(LC_50_)^b^, µM			
Human erythrocytes	≫250	≫250	≫250	≫250

^a^Bovine myeloid antimicrobial peptide-27 (BMAP-27)

^b^50% cell death compared to untreated cells

Hemolysis was determined by incubating a 10% (vol/vol) suspension of human erythrocytes with peptides. Erythrocytes were rinsed (×4) in 10 mM PBS, pH 7.2, by repeated centrifugation and re-suspension (3 min, 3000×*g*). The cells were then incubated at room temperature for 1 h in either deionized water (fully hemolyzed control), PBS, or peptide in PBS. After centrifugation (5 min, 10,000×*g*), the supernatant was separated from the pellet, and the absorbance was measured at 550 nm. Absorbance of the suspension treated with deionized water determined complete hemolysis. The values given in Table 1 are concentrations needed to kill half of the sample population (50% lysis of human erythrocytes) and are expressed as median lethal concentrations (LC_50_). All tests were done in triplicate.

## Results

To probe folding-mediated antimicrobial interactions, a model peptide, KARLAKLRARLYRLKARLARL, was designed. The sequence consists of three generic heptads, *PPPHPPH*, in which *P* is polar or small (alanine) and *H* is hydrophobic. The heptads and their continuity in the sequence ensure that *i* and *i* + 7 residues are placed next to each other when viewed along the helical axis, and therefore can be of the same type (Akerfeldt et al. 1993). This feature ensures that hydrophobic and polar residues can segregate, upon formation of alpha helical conformation, onto distinct faces of one amphipathic helix. To avoid hemolytic activities common for venom peptides, which tend to have extended hydrophobic faces (Ryadnov et al. 2009), the hydrophobic face of the model peptide was held to the 1:1.5 ratio of hydrophobic to cationic residues. To constrain this ratio, small and neutral alanines, which are not involved in membrane binding, were used in the polar face as a neutral, solvated cluster. The combination of these design principles ensures the folding of a contiguous amphipathic helix in phospholipid membranes (Rakowska et al. 2013). Another peptide, QAELAQLEAQLYELQAELAEL, was designed as an anionic counterpart of the antimicrobial sequence. This peptide has similar propensities for helical folding, it is negatively charged, should not bind to membranes, and it should not be biologically active. Therefore, it is meant as a control, both structural and functional. Both peptides incorporate a single tyrosine residue as a reporter for structural measurements. For the purpose of this study, the two synthetic peptides were termed as (+)-helix (antimicrobial and cationic) and (−)-helix (non-antimicrobial and anionic).

Consistent with the design rationale, (+)-helix exhibited strong antimicrobial activities that were comparable with those of naturally occurring AMPs, such as cecropin B and cathelicidin 27, while showing no hemolytic activity (Table 1). As expected, (−)-helix was not biologically active.

Most bacterial membranes tend to maintain the high content of anionic phosphatidylglycerol (PG) components, which render them negatively charged. The negative charge of PG is distributed in the membranes by neutral lipids. In different bacteria, these neutral lipids can vary. For example, *P*. *aeruginosa* mixes PG with phosphatidylcholine (PC) and phosphatidylethanolamine (PE) lipids (Sohlenkamp and Geiger 2016), while *S*. *aureus* lacks PE completely (Epand et al. 2009).

Cell lipids are also typically unsaturated. However, to better understand whether the antimicrobial effect of the antimicrobial peptide depends on the nature of the lipid, we used two types of lipids: a saturated short-chained and an unsaturated long-chained ones to assemble fluid lipid bilayers. For both types, bacterial mimetic membranes contained PG. Specifically, short-chain and saturated neutral lipid dilauorylphosphatidylcholine (DLPC) (C12) was used to form thin neutral membranes and its 3:1 mixtures (molar ratios) with anionic dilauroylphosphatidylglycerol (DLPG) assembled into anionic (bacterial) model membranes (Opdenkamp 1979). Thicker membranes were composed of longer (C18) and unsaturated neutral lipid palmitoyl oleoyl phosphatidylcholine (POPC), to give neutral membranes, and its 3:1 mixtures (molar ratios) with palmitoyl oleoyl phosphatidylglycerol (POPG) to provide bacterial mimetics. All four formulations were assembled as large unilamellar vesicles (LUVs) of around 100 nm in diameter.

### Sizing

#### Dynamic light scattering

Dynamic light scattering (DLS) was used to confirm the size and homogeneity of prepared LUVs after extrusion, and to monitor changes in the hydrodynamic radii of each model as a function of peptide concentration. The size of bare liposomes was compared with the size of complexes formed after introducing peptide at different lipid-to-peptide (L/P) mole ratios (Table 2). No significant changes were observed for POPC/PG, POPC, and DLPC using (+)-helix, while increases to ~150 nm were evident for mixtures of DLPC/PG with (+)-helix. The addition of the anionic (−)-helix to the charged DLPC/PG and POPC/PG had no effect on size changes. The results are consistent with the preferential binding of (+)-helix with anionic membranes and suggest stronger interactions with thinner membranes. Indeed, further (+)-helix additions revealed two ratio cut-offs at which LUV of both types collapse as judged by larger sizes and broader size distributions–a 40:1 L/P ratio for POPC/PG and a 20:1 L/P ratio for DLPC/PG.

**Table 2:** Z-average particle diameters measured by DLS for all four LUVs prepared alone and with peptides at different L/P mole ratios

	Liposome alone	100:1	60:1	40:1	20:1
POPCPG/(+)-helix	124 (0.10)	123 (0.10)	120 (0.10)	HS	HS
DLPCPG/(+)-helix	108 (0.09)	134 (0.09)	137 (0.09)	149 (0.11)	HS
POPCPG/(−)-helix	124 (0.10)	124 (0.09)	124 (0.08)	123 (0.10)	123 (0.09)
DLPCPG/(−)-helix	108 (0.09)	111 (0.08)	111 (0.08)	112 (0.10)	112 (0.08)
POPC/(+)-helix	156 (0.10)	154 (0.09)	152 (0.09)	154 (0.10)	154 (0.08)
DLPC/(+)-helix	107 (0.07)	107 (0.07)	106 (0.09)	106 (0.07)	107 (0.06)

Lipid concentration was kept constant at 1.2 mM. Each measurement was done in triplicate and the data shown is an average with an error of 2%. The mean PDI is shown in* brackets*. High scattering (HS) reflects high polydispersity

### Correlative characterization of peptide and lipid structuring

Peptide concentration and ratio dependences revealed by DLS suggest that (+)-helix interacts with negatively charged membranes. To gain a better insight into this, peptide folding with and without membranes was probed using fluorescence, CD, and NMR spectroscopies.

Fluorescence spectroscopy provides a straightforward and precise measure of peptide interactions in given environments based on fluorescence emission of aromatic amino acids. Changes in intrinsic fluorescence are used to monitor structural changes in polypeptides or to detect intermolecular interactions. Thus, for both peptides, tyrosine fluorescence was used to monitor lipid–peptide interactions.

**Fig. 1:**
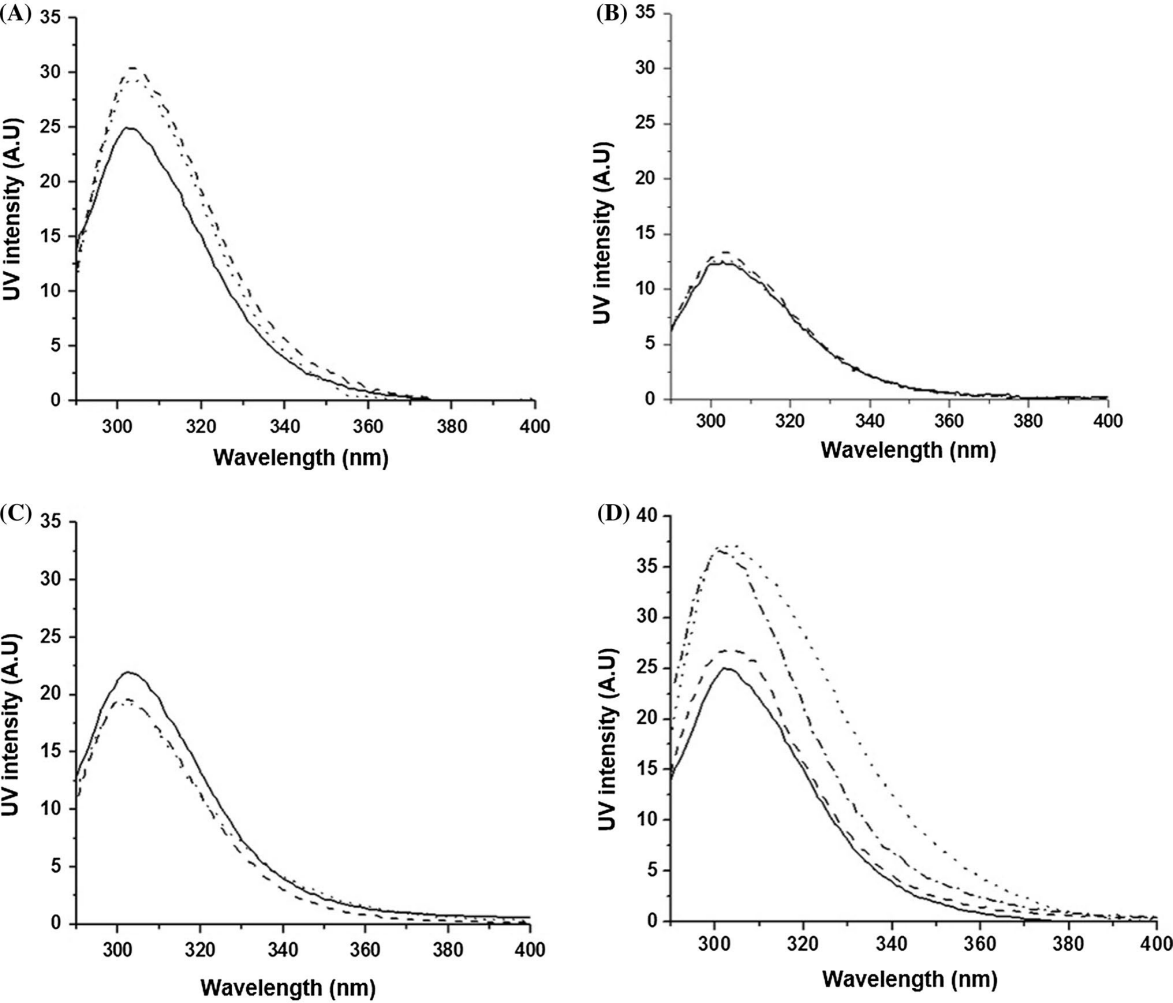
Fluorescence spectra of: **a** individual (+)-helix (15 µM) (*solid line*) and in the presence of POPC/PG (*dotted line*) and DLPC/PG (*dashed line*); **b** (+)-helix (10 µM) alone (*solid line*) and in the presence of POPC (*dotted line*) and DLPC (*dashed line*); **c** (−)-helix (12 µM) alone (*solid line*) and in the presence of POPC/PG (*dotted line*) and DLPC/PG (*dashed line*); **d** (+)-helix (15 µM) in phosphate buffer solution (*solid line*) and TFE 50% (*dotted line*); (−)-helix (15 µM) in phosphate buffer solution (*dashed line*) and in TFE 50% (*dashed*–*dotted line*). Conditions: phosphate buffer 10 mM, pH 7.4; 100 L/P mole ratio. The signals of liposomes DLPC/PG, POPC/PG, DLPC, and POPC alone in phosphate buffer were subtracted from the relevant fluorescence spectra

Fluorescence spectra for (+)-helix show increased intensities in the presence of anionic LUVs when compared to those for free peptide (Fig. 1a). The intensity proved to be the same for all the L/P ratios tried at which no precipitation occurred (i.e., 100, 80, 60) (data not shown here).

In contrast, no changes were found for (+)-helix in the presence of neutral LUVs (Fig. 1b), which is in good agreement with the DLS data. In addition, no changes in fluorescence were recorded for (−)-helix in the presence of anionic LUVs (Fig. 1c).

Further, the fluorescence of both peptides in phosphate buffer and 50% aq. trifluoroethanol (TFE) was compared to test whether the observed changes are related to a specific tyrosine environment, i.e., more hydrophobic or hydrophilic. Fluorinated alcohols promote intramolecular hydrogen bonding by excluding water from the solute, which they achieve by encompassing peptides in a hydrophobic “matrix” and lowering the dielectric constant. Thus, the secondary structure observed in the presence of TFE would correspond to that of the peptide alone defined by the helical propensity of its backbone (Buck 1998; Lee et al. 1999; Roccatano et al. 2002; Wang et al. 2014). As expected, upon excitation at 280 nm, both peptides exhibited fluorescence emission maxima at 303 nm. The intensity of the fluorescence for both peptides increased upon the TFE addition (Fig. 1d). These increases in fluorescence observed for the tyrosine in (+)-helix suggest that the peptide folds into an α-helical structure in anionic LUVs (Chen and Barkley 1998; Tan et al. 2014). To support this conjecture, helical folding was assessed using CD spectroscopy.

**Fig. 2:**
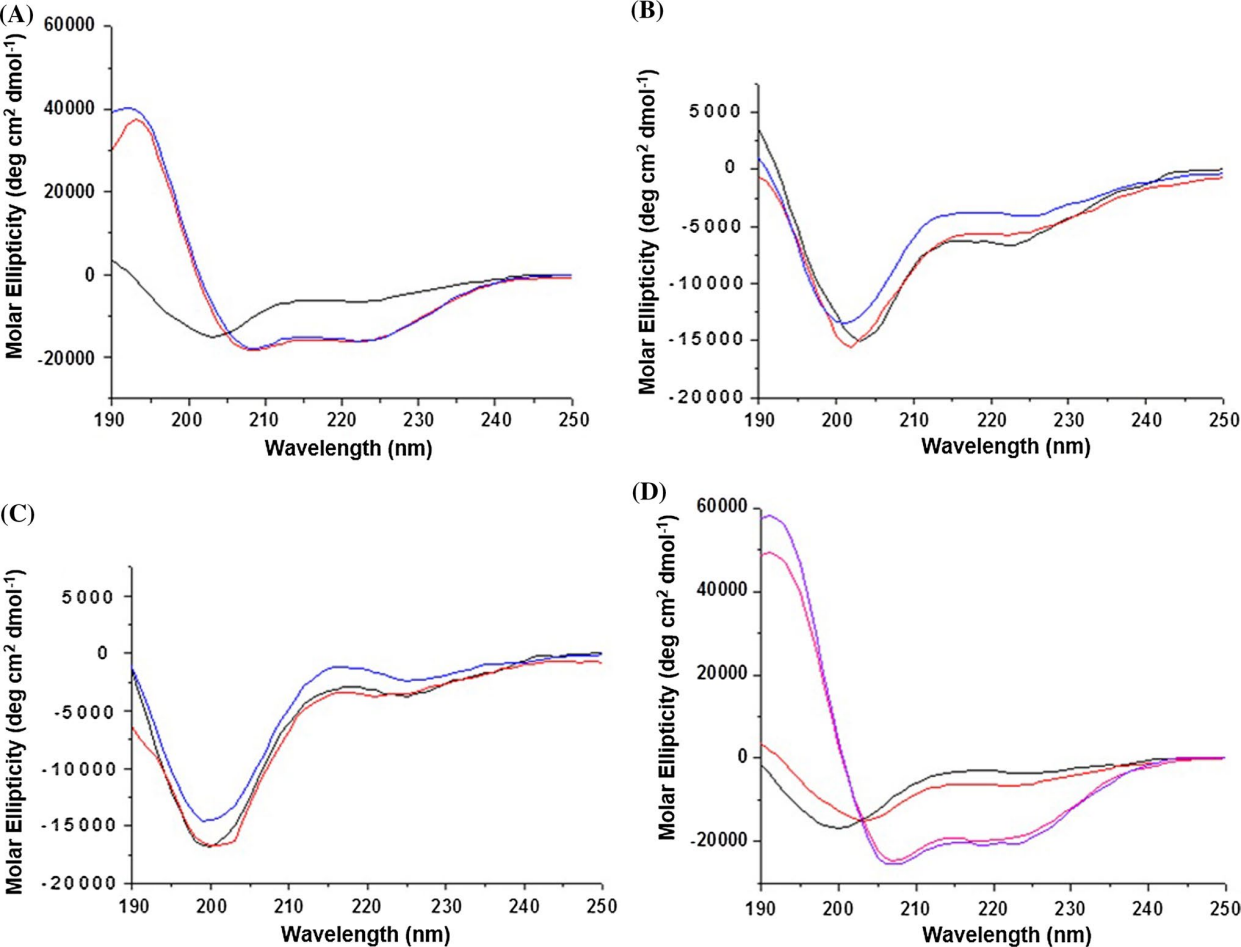
CD spectra of: **a** (+)-helix peptide alone (*black*) and in the presence of POPC/PG (*red*) and DLPC/PG (*blue*); **b** (−)-helix peptide alone (*black*) and in the presence of POPC/PG (*red*) and DLPC/PG (*blue*); **c** (+)-helix alone in (*black*) and in the presence of POPC (*red*) and DLPC (*blue*); **d** (+)-helix alone in buffer phosphate (*red*) and in 50% aq TFE (*pink*), (−)-helix alone in buffer phosphate (*black*) and in 50% aq. TFE (*purple*). Conditions: 10 mM phosphate buffer, pH 7.4; 100 L/P mole ratio

CD spectra for (+)-helix alone were characteristic of a random coil conformation (Fig. 2a). In contrast, CD spectra for the peptide in the presence of anionic LUVs were indicative of appreciable α-helical conformations with a typical maximum at 195 nm and two minima at 208 and 222 nm. Consistent with the DLS data, CD spectra recorded at different L/P ratios revealed two cut-offs at 50 and 30 for PO and DL anionic types, respectively, below which decreases in helical signals were apparent, suggesting membrane collapse and lipid precipitation (data not shown). As expected, CD spectra for (−)-helix for anionic LUV and for (+)-helix for neutral LUVs showed random-coil conformations (Fig. 2b, c), indicating that no lipid–peptide interactions took place.

The results together indicate that (+)-helix exhibits folding-mediated binding to anionic membranes with the formation of α-helical structures at the L/P ratios of 100–30, while its anionic counterpart does not fold at any conditions used. To confirm that the folding of (+)-helix is mediated by membrane binding; the peptides were tested in 50% aq. TFE (Fig. 2d). Both (+)-helix and (−)-helix have similar helical propensities, and differ only by their ability to bind to anionic membranes in a folding-responsive manner. Gratifyingly, CD spectra obtained in 50% aq. TFE for both peptides were expectedly helical, thus indicating membrane-dependent (+)-helix folding. Further support for this came from ^1^H-nuclear magnetic resonance (^1^H-NMR) measurements, which provide a straightforward probe of primary and secondary polypeptide structure (Case 2000).

**Fig. 3:**
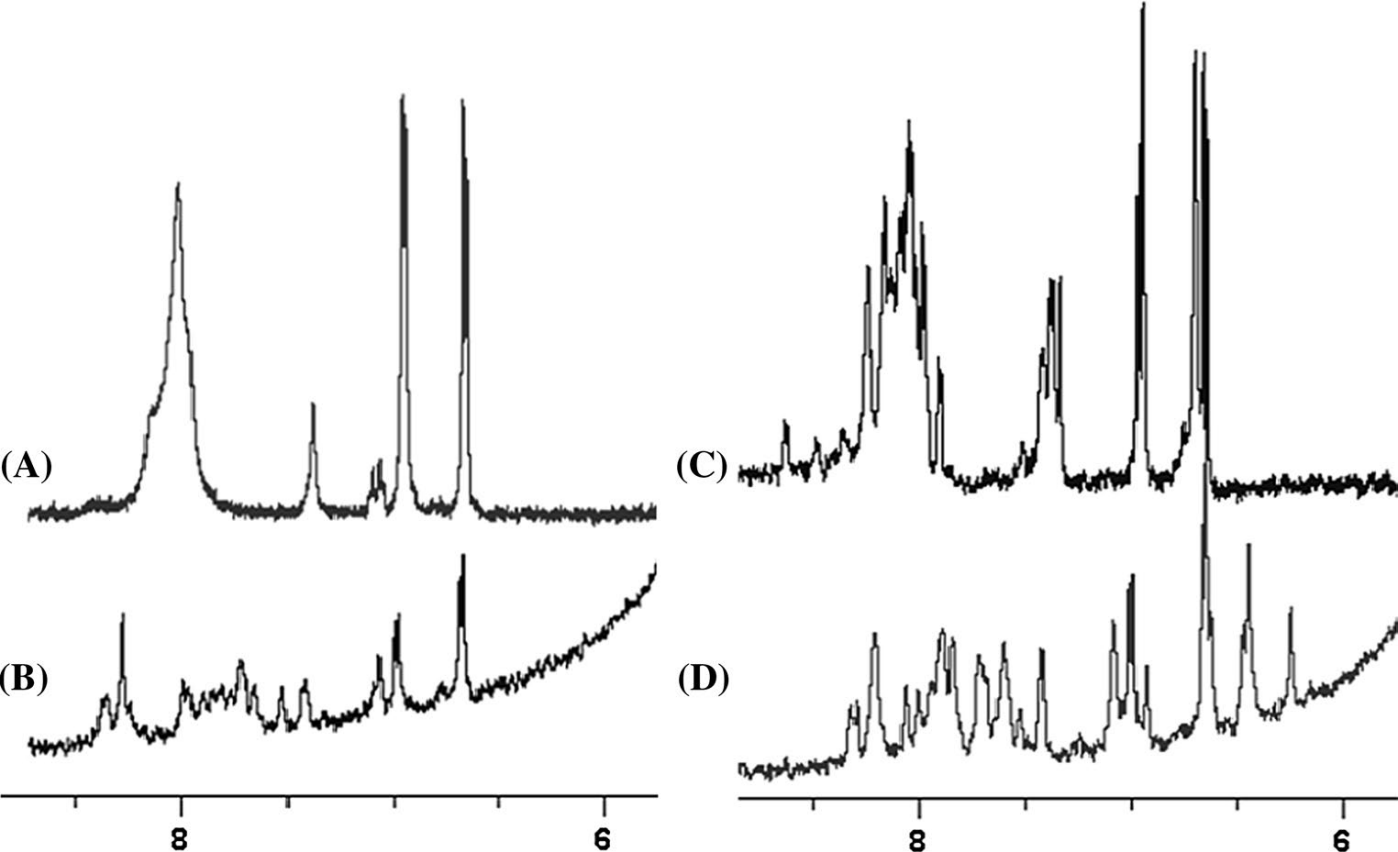
^1^H-NMR spectra of (+)-helix (12 µM) in **a** phosphate buffer and **b** 50% aq. TFE; for (−)-helix (12 µM) in **c** buffer and **d** 50% aq. TFE

^1^H-NMR spectra for both peptides in phosphate buffer gave two doublets at 6–7 ppm corresponding to aromatic (tyrosine) hydrogens and a broad peak at ~8 ppm characteristic of degenerate amide protons (Fig. 3a, c). This is in good agreement with random-coil conformations occupying the same spectral region of 6–9 ppm, within which substantial peak overlapping is allowed (Wüthrich 2001). Consistent with the results obtained by CD and fluorescence spectroscopies, signal dispersions suggesting secondary structure formation were apparent for both peptides in 50% aq. TFE (Fig. 3b, d).

**Fig. 4:**
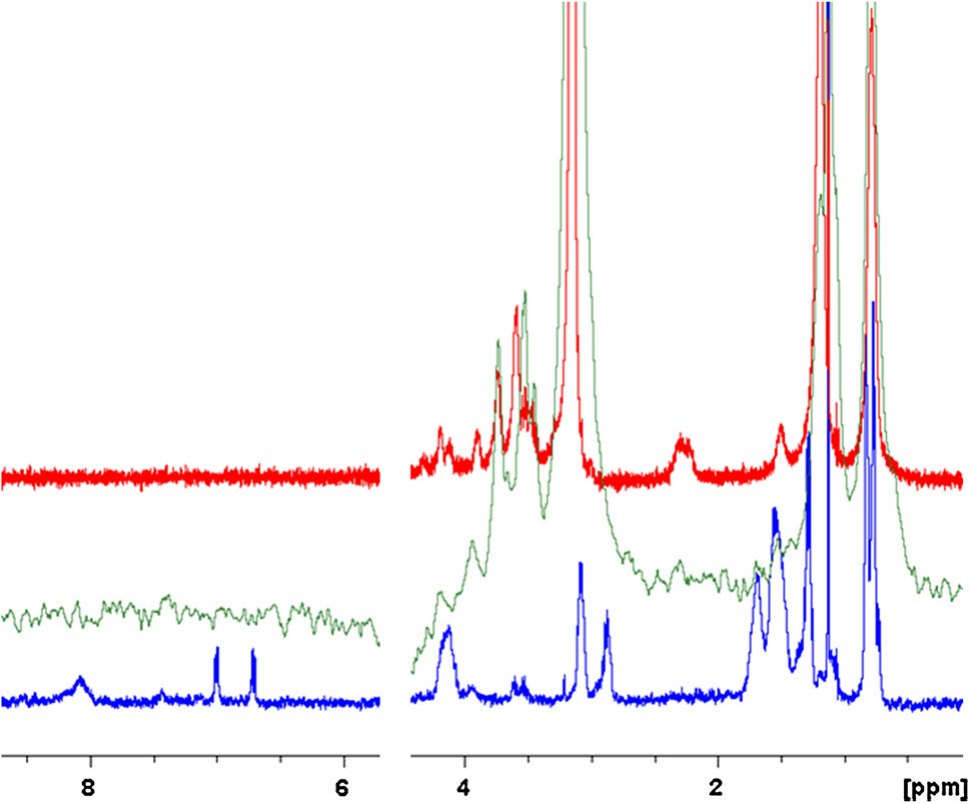
^1^H-NMR spectra of POPC/PG (1.2 mM) (*green*), (+)-helix (12 µM) in buffer (*blue*) and in the presence of POPC/PG (0.72 mM) at L/P 60:1 (*red*)

We then used NMR to detect the structure of (+)-helix bound to liposomes. Figure 4 shows the spectra of free liposomes (red), of free (+)-helix (blue), and of the POPC/POPG (3:1)-(+)-helix complex at 60:1 ratio (green). The NMR spectrum of the complex does not show any signal from (+)-helix in the N–H region, but shows line broadening for the lipid peak at 3.35 ppm assignable to the terminal N(CH_3_)_3_ moiety of the choline group (Cruciani et al. 2006).

The absence of ^1^H signal from the peptide in the POPC/PG-(+)-helix complex could be due both to the heterogeneity of the system and to the large increase in line width of the peptide signal upon binding to the liposome. In fact, the liposome has a measured hydrodynamic diameter of around 120 nm, and an estimated molecular weight of 60,000 kDa and correlation time in the microsecond range (Mouret et al. 2014), which, compared to the nanoseconds range for the free peptide, leads to extreme broadening for the peptide signals in the liposome–peptide complex.

In the NMR spectra of the lipid, the peaks corresponding to the choline head group (at 3.35 ppm) and the terminal methyl of the alkyl chain (at 0.98 ppm) are apparent and well separated, and their full width at a half height can be measured. The head choline group shows an increase in line width from 6.7 Hz for the free liposome to 11.8 Hz for the complex. On the contrary, the terminal methyl group shows only a small increase from 19.3 to 21.5 Hz. The line broadening of the choline head group in the complex is consistent with the increased heterogeneity of the complex (when compared to the liposomes alone) and decreased mobility of the group due to strong interactions with the peptide.

These results, together with appreciable helix formation upon interaction with liposomes, support membrane-mediated peptide folding.

## Discussion

The objective of this study was to probe tertiary peptide–membrane contacts that are perceived as characteristic of membrane-mediated antimicrobial activity. To achieve this, we have interrogated a pair of rationally designed peptides, antimicrobial and inactive forms, based on the same sequence template.

Using two types of reconstituted model membranes mimicking bacterial and mammalian membranes, respectively, which were assembled from two different phospholipid compositions, saturated and unsaturated lipids, we have performed a comparative assessment of these interactions for both active and inactive peptide forms, i.e., (+)-helix and (−)-helix. Within each type, the distinction was made as to the membrane thickness to enable two-dimensional measurements of (1) peptide folding, in response to lipid binding, and (2) lipid behavior in contact with peptide including membrane collapse and bilayer fluctuations.

The study confirmed the overarching concept of antimicrobial peptides being able to induce membrane perturbations and subsequent disintegration by incorporating into anionic phospholipid membranes. ^1^H-NMR spectra suggested interfacial peptide binding at the expense of decreased mobility of phospholipid headgroups in the anionic and relatively rigid PO membranes. This was compounded by appreciable lipid-signal broadening in ^1^H-NMR spectra indicating heterogeneity of peptide–lipid interactions, which proved to be dependent on L/P ratios. Consistent with the NMR measurements, DLS spectra revealed strong correlations between the binding of (+)-helix and the L/P ratios with lower ratios leading to increased polydispersity of LUVs and lipid aggregation. Notably, cut-off L/P ratios of 40 and 20 for anionic PO and DL types were determined, which were in good agreement with results from conformational studies by CD and fluorescence spectroscopies confirming that changes in membrane structure were a direct result of membrane-mediated peptide folding.

The same measurements using zwitterionic membranes gave no apparent responses, indicating that membrane-binding is a synergistic interplay of interfacial electrostatic and hydrophobic interactions. As expected, comparative measurements under the same conditions for (−)-helix gave no evidence of peptide–lipid binding, while nearly identical TFE-promoted conformational responses for both peptides confirmed their nearly identical helical propensities.

All in all, the obtained results indicate charge-dependent and folding-mediated antimicrobial interactions at peptide–lipid interfaces, while being strongly supportive of multi-step peptide incorporation into lipid bilayers. The study provides a straightforward approach for modulating structure–activity relationships in the context of membrane-induced antimicrobial action, thus holding promise for the rational design of potent antimicrobial agents.
